# Combined Use of Transcutaneous Electrical Nerve Stimulation and Short Foot Exercise Improves Navicular Height, Muscle Size, Function Mobility, and Risk of Falls in Healthy Older Adults

**DOI:** 10.3390/ijerph19127196

**Published:** 2022-06-11

**Authors:** Juntip Namsawang, Pornpimol Muanjai

**Affiliations:** 1 Department of Physical Therapy, Allied Health Sciences Faculty, Burapha University, Chonburi 20131, Thailand; juntip@go.buu.ac.th; 2 Exercise and Nutrition Innovation and Sciences Research Unit, Burapha University, Chonburi 20131, Thailand

**Keywords:** electrical stimulation, intrinsic foot exercise, abductor hallucis muscle, cross-sectional area, functional test, elderly

## Abstract

Electrical stimulation is an established method that is used to improve muscle strength. The present study compared changes in the navicular drop test (NDT), muscle size, the five times sit to stand (5TSTS) test, the timed up and go (TUG) test, and the risk of falls in response to transcutaneous electrical nerve stimulation (TENS) plus short foot exercise (SFE) and SFE alone in 68 healthy elderly participants aged 65–75 years. Participants were randomly assigned to two groups: TENS plus SFE and SFE alone (with sham TENS). Measurements of NDT, muscle size, 5TSTS, TUG, and risk of falls were made before and after 4 weeks of training. The NDT was significantly improved by a median of 0.31 mm in the TENS plus SFE group and 0.64 mm in the SFE alone group (*p* < 0.001). Similarly, there was a significant improvement in Falls Efficacy Scale International (FES-I), 5TSTS, and TUG for both groups (*p* < 0.001). The abductor hallucis muscle size increased by 0.23 cm^2^ in the TENS plus SFE group and 0.26 cm^2^ in the SFE alone group (*p* < 0.001). There were no significant differences between the two groups for any variables (*p* > 0.05) except TUG, which showed a greater improvement in the TENS plus SFE group (*p* = 0.008). Our findings demonstrated that TENS plus SFE and SFE alone improved intrinsic foot muscle size. However, TENS plus SFE tended to improve NDT more than SFE alone, particularly in cases of severe muscle weakness. Thus, the combined use of TENS plus SFE could be recommended for muscle strengthening and balance programs for fall prevention in older adults.

## 1. Introduction

Falls are a serious concern worldwide and lead to health problems or diseases. They are the second leading cause of death in the older adult population [1,2], affecting 28–35% of people aged ≥65 years and 32–42% of those aged >70 years [3]. After a fall, patients frequently fear subsequent falls, which can lead to loss of confidence, reduced physical and social activities, depression, loss of mobility, increased risk of falls, physical weakness, and a strong negative impact on quality of life in older people [4]. A fear of falling occurs in 26–55% of the aging population. Several factors lead to falls and fear of falling, such as, old age, previous history of falls, poor health, and decreased physical function or mobility [4].

There is a lot of evidence supporting the association between falls and muscle weakness. Moreland et al. [5] showed that older adults with lower limb muscle weakness had a higher rate of falls (odds ratio (OR), 1.76; 95% confidence interval (CI), 1.31–2.37) and recurrent falls (OR, 3.06; 95% CI, 1.86–5.04). Mickle et al. [6] showed that foot muscle weakness was an independent predictor of falls in older adults and the weakness of this muscle further affected balance control in dynamic circumstances and a reduced generation of propulsive power [7,8,9]. Specifically, the abductor hallucis (AbdH) muscle belongs to the plantar group of intrinsic foot muscles that act as an active subsystem providing dynamic stability of the medial longitudinal arch (MLA). The AbdH muscle originates and inserts within the foot and the reduced function of this muscle can lead to foot instability, MLA malalignment, and postural instability [10,11]. McKeon et al. [12] reported their concept of the foot core system that further refined the foot structure into active, passive, and neural subsystems. Moreover, Fiokowski et al. [13] and Headlee et al. [14] showed that blocking tibial nerve transmission led to functional impairment of the AbdH muscle and exercise-induced muscle fatigue increased the navicular drop. The navicular drop provides a good indicator of the function of the intrinsic foot muscles during weight bearing (R = 0.47, *p* = 0.03) [14] and weakness of the AbdH in older adults has been associated with increased risk of falls [6]. Park et al. [15] also showed that individuals with foot muscle impairment had a fourfold higher risk of falls.

A previous sonographic study showed that the cross-sectional area (CSA) of the AbdH muscle may be a useful early biomarker for foot muscle weakness [16]. In addition, AbdH muscle weakness was related to decreased CSA [17]. Jandova et al. demonstrated a significant reduction in strength, CSA, and foot muscle thickness in healthy older adults [18]. A reduced muscle size is closely associated with a loss of muscle strength related to sarcopenia. A 30–57% reduction in muscle size was found in older compared with younger adults, which may affect walking and balance in older people [19]. Functional performance, measured by the timed up and go (TUG) test and the five times sit to stand (5TSTS) test, is widely used to evaluate functional mobility in older adults. In particular, the TUG test has been reported to be strongly related to the mobility status of the elderly and is a useful tool for movement impairment prevention, whereas 5TSTS can be used to identify poor exercise tolerance and impaired functional performance and is a useful outcome measure in rehabilitation [20].

Therefore, exercise-induced muscle strengthening of the AbdH muscle is important to prevent or delay the risk of falls in older adults and can be achieved in many ways, such as foot manipulation with paper, short foot exercise (SFE), and electrical stimulation (ES) [8]. SFE is a strengthening exercise that can be easily and safely performed. It is specific to targeted intrinsic foot muscles and does not recruit the extrinsic foot muscles. A previous study showed an increase in navicular height after SFE training [21]. Lee et al. [22] found greater improvements in proprioception, dynamic balance, and the ankle stability index in individuals with ankle instability following an 8-week SFE training compared with traditional proprioceptive sensory training. Lynn et al. [23] also showed improved postural stability as measured using a dynamic balance test during dynamic activity after a 4-week SFE training program.

Transcutaneous electrical nerve stimulation (TENS) is a type of ES method that is readily available, inexpensive, and relatively easy to use as a part of a rehabilitation program or in everyday life. It is an effective modality to enhance foot muscle strength and balance in older stroke patients [24]. There is additional evidence that patients with knee osteoarthritis have increased muscle strength and a beneficially altered gait following therapeutic exercise in conjunction with TENS [25]. We have previously reported that TENS enhanced navicular height [8] and static balance, and reduced the fear of falling in elderly people after 4 weeks. AbdH muscle activity and CSA were substantially increased following 4 weeks of neuromuscular electrical stimulation (NMES) together with SFE in young adults with flat feet [26].

The effects of TENS with SFE in older adults on muscle size adaptation, balance, and risk of falls remains unclear. Therefore, the present study compared the effects of TENS combined with SFE and SFE alone for 4 weeks on the navicular drop test (NDT), CSA of the AbdH muscle, TUG, 5TSTS, and risk of falls assessment. We hypothesized that the CSA of the AbdH muscle would be significantly greater following TENS with SFE compared with SFE alone and that other outcomes would show greater changes in response to the combined intervention compared with SFE alone.

## 2. Materials and Methods

### 2.1. Participants

A total of 68 sedentary older adults (aged 65–75 years) were recruited from Chonburi Province, Thailand, and randomly assigned to two groups using block randomization (4 × 4). The characteristics of the study participants in the two groups are shown in Table 1. The inclusion criteria were participants with body mass index (BMI) < 30 kg/m^2^, able to communicate or follow instructions, independently living, able to maintain the standing position, and able to walk independently without mobility aids. The exclusion criteria were using a wearable pacemaker, history of foot wound, foot or ankle pain, foot surgery or amputation, broken or irritated skin at the electrode site, neurological disorders, or falls within the past 6 months. The study conformed to the standards set out by the most recent revision of the Declaration of Helsinki and was approved by the Research and Innovation Administration Division of Burapha University Ethics Committee (registration number: IRB-No. 272/2562).

### 2.2. Study Design

**Figure 1:**
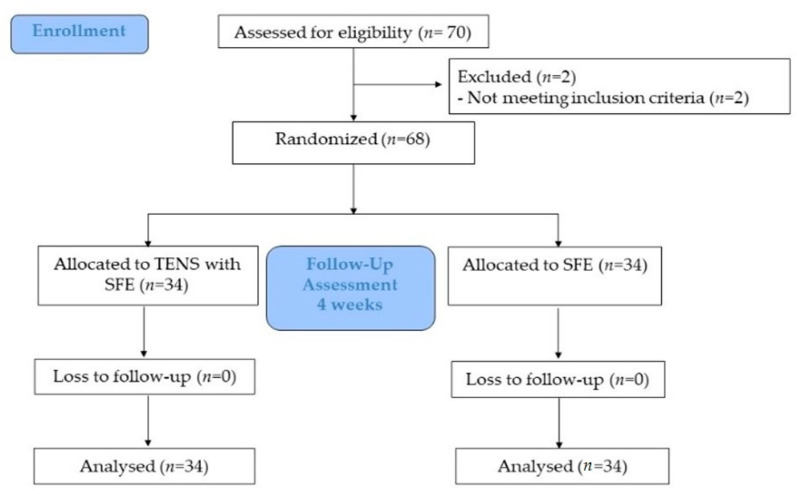
Flow chart of the participants. TENS, transcutaneous electrical nerve stimulation; SFE, short foot exercise.

The present study was a double-blinded randomized controlled trial (Figure 1). Prior to taking part in the study, participants visited our laboratory to take part in a familiarization session with the procedures, tools, and measurements, and a pretest was conducted a few days later. Measurements of NDT, CSA of the AbdH muscle, TUG, 5TSTS, and Falls Efficacy Scale International (FES-I) were made prior to the intervention and again 4 weeks later. Each participant was randomly assigned to one of two groups and received either TENS plus SFE or SFE with sham TENS for 3 days per week for 4 weeks. All tests were performed by the same person and used the dominant foot.

### 2.3. Intervention

SFE was performed in both groups. Participants performed SFE in a standing position and attempted to pull the head of the metatarsal bones toward the calcaneus, generating maximum contraction of intrinsic foot muscles, without bending the distal phalanx. Lifting of the forefoot or sole was carefully controlled, held for 5 s, and relaxed for 30 repetitions per day, 3 days per week, for 4 weeks. ES intervention was performed using an asymmetrical biphasic square waveform of TENS current (a pulse duration of 200 µs with a frequency of 100 Hz) [8,24,27,28] with a 38-mm diameter of electrodes attached over the AbdH muscle belly. The active electrode was placed over the motor point, while the dispersive electrode was placed behind the head of the first metatarsal bone. The maximum intensity of the TENS current seen with tetanic contraction was released until the participant felt maximal toleration without pain or uncomfortable feeling in the TENS plus SFE group, whereas the electrical current was not given (0 mA) to the SFE alone group. The intervention lasted 30 min per session, with 3 sessions per week for 4 weeks.

### 2.4. Outcome Measures

NDT was measured as the distance from the navicular tuberosity to the floor level using a digital vernier caliper (570 series; Mitutoyo Co., Kanagawa, Japan) while the feet were bearing and not bearing weight. First, NDT was first defined in the sitting position (non-weight-bearing) with the hip and knee flexed at 90° and the feet aligned in the neutral position, and the navicular height distance was noted. Second, the navicular height distance was repeatedly measured with the participants standing while bearing their weight equally between both feet and with the full knee extension regardless of any foot contraction. Three measurements were recorded in each position for further analysis, and the difference in the average values between positions was reported as the NDT value. The reliability of the measurement was very high (ICC_3,1_ = 0.98).

Ultrasound imaging (M5 series; Shenzhen Mindray Bio-Medical Electronics Co., Shenzhen, China) with a 38-mm aperture and 7.5-MHz linear array probe (7L4s, Shenzhen Mindray Bio-Medical Electronics Co., China) was used to detect the CSA of the AbdH muscle while participants were lying down with a small pillow supported under the knee, which was at 15 degrees of flexion and the foot positioned in the anatomical position. An ultrasound probe was placed perpendicular to the AbdH muscle 1 cm behind the navicular tuberosity with minimum pressure on the tissue [26]. Three images of the AbdH muscle were captured in this view and analyzed using ImageJ version 1.8.0 (National Institutes of Health, Bethesda, MD, USA). The average of the three images was used for further analysis. The reliability of the measurement was high (ICC_3,1_ = 0.90).

5TSTS was indicated to lower limb muscle strength and balance control wherein five measurements of sitting in a standard chair (height, 43 cm; depth, 47.5 cm) to fully standing as fast as possible for five repetitions without back resting on the back of the chair [29,30]. To perform this test, the participants began sitting in a chair, with arms crossed over their chest and their back resting on the back of the chair. The time spent was measured thrice with a 1-min rest. The average of the three repetitions was considered for further analysis [30].

In this study, to determine the functional mobility and balance ability, the TUG test was used, which is recommended for routine screening for falls, in the guidelines published by the American Geriatric Society and the British Geriatric Society [31]. The time recorded in the TUG test more than or equal 13.5 s was considered as the cut off for those at risk for falls [32]. The participants were asked to walk as fast as possible, and the time taken to stand up from the chair and walk straight for 3 m, turning to the chair (approximate seat height, 46 cm) was recorded [32]. The test was repeated thrice with a 1-min rest between the tests, and the average time was used for further analysis. Excellent test-retest reliability was represented in older adults (ICC = 0.99) [33].

In this study, fear of falling was assessed using FES-I questionnaire in the Thai language (Cronbach’s alpha = 0.95) [34]. The Thai version of the form comprised 16 ability-related questions, including physical and psychosocial performance, scored using a 4-point Likert-type scale, where “1” indicated no fear at all and “4” indicated the maximum fear of falling. A total of 16–21 points represented no fear of falling, 22–27 points indicated mild to moderate fear of falling, and 28–64 points indicated the highest fear of falling [8,34].

### 2.5. Statistical Analysis

The sample size was calculated using a power of 0.90, alpha error of 0.05, and effect size of 0.5 (G * Power 3.0.10, Franz Faul, University of Kiel, Kiel, Germany) [35]. A sample size of 30 participants per group was estimated to be necessary. This size was increased to compensate for any alterations in the statistical significance of the results due to dropout, and a total of 34 participants were recruited for each group. Descriptive data were presented as mean ± SD and were tested for normality using the Shapiro–Wilk test. None of the variables were normally distributed; therefore, a non-parametric statistical analysis was used. The changes in all variables between the two groups were then presented as the median and interquartile range (IQR). The Wilcoxon sign rank test was used to compare changes within groups, and the Mann–Whiney U test was used for comparisons between groups. An alpha level of 0.05 was used to determine the statistical significance. A linear least squares linear regression was used to determine the relationship between changes in NDT score before and after training. All statistical analyses were performed using Statistic Package for the Social Sciences (SPSS for Windows version 24.0, Chicago, IL, USA).

## 3. Results

All participants completed all the training sessions 3 days per week for 4 weeks. No adverse effects were reported during the training sessions. The baseline measurements of all variables for the two groups are presented in Table 1 and showed no differences between the two groups (*p* > 0.05).

Table 2 shows the changes in all variables after 4 weeks of training. The NDT, measured using a digital vernier caliper, was significantly improved by a median of 0.31 mm (−0.00, 0.98 mm) in the TENS plus SFE group and 0.64 mm (0.04, 1.14 mm) in the SFE group (*p* < 0.001). There was a similar improvement in FES-I score, which substantially decreased by a median of two points (one, four points) for both groups (*p* < 0.001). An increase in the AbdH muscle size was also shown, with median increases of 0.23 cm^2^ (0.09, 0.40 cm^2^) in the TENS plus SFE group and 0.26 cm^2^ (0.15, 0.37 cm^2^) in the SFE group (*p* < 0.001). Similarly, there was an improvement in the 5TSTS results, with a median of 0.52 s (0.21, 1.42 s) in the TENS plus SFE group and 0.96 s (0.16, 1.30 s) in the SFE group (*p* < 0.001).

TUG was improved by a median of 0.94 s (0.55, 1.14 s) in the TENS plus SFE group and 0.53 s (0.31, 0.91 s) in the SFE group (*p* < 0.001). There were no significant differences between the two groups for any of the variables (*p* > 0.05) except TUG, which showed a greater improvement in the TENS plus SFE group than the SFE group (*p* = 0.008).

**Figure 2:**
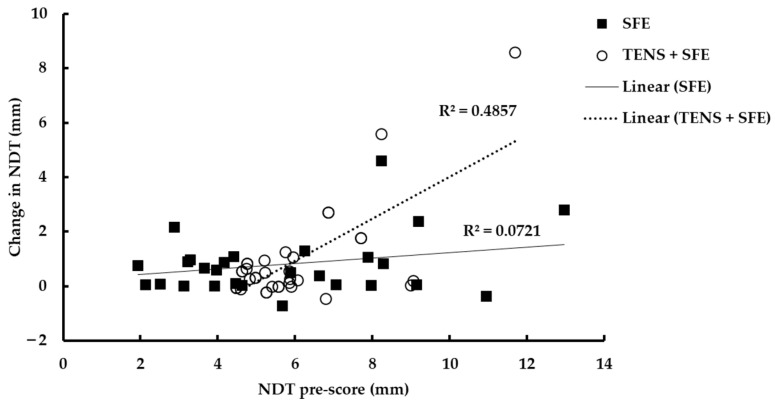
Changes in NDT score as a result of 4 weeks training in relation to the NDT baseline score. Solid line and symbols, SFE group; dashed line and open symbols, TENS + SFE group.

Although the improvement in NDT after 4 weeks of training was related to the baseline values of NDT, participants with the greatest severity of NDT (a higher NDT) showed larger improvements than those with less severity (a stronger AbdH muscle) (Figure 2; R^2^ = 0.49, *p* < 0.001) in the TENS plus SFE group, although this was not seen in the SFE group. We examined whether these differences in the severity of the NDT value in the TENS plus SFE group were reflected in the other variables in this study. We compared 17 older adults with the lowest NDT scores with 17 older adults with the highest NDT scores and found no differences in the TUG, FES-I, CSA of AbdH muscle, or the 5TSTS after 4 weeks of training between the two groups according to NDT score (*p* > 0.05).

## 4. Discussion

The present study examined the effects of combined use of TENS and SFE on the NDT, CSA of the AbdH muscle, TUG, 5TSTS, and FES-I in healthy older adults. Our results demonstrated that the TENS plus SFE and SFE alone groups showed significant improvements in the NDT, TUG, 5TSTS, and FES-I. Both groups also showed a significant increase in the CSA of the AbdH muscle. However, the change in TUG was significantly greater in the TENS plus SFE group than the SFE alone group.

NDT was substantially decreased in the TENS plus SFE group after 4 weeks. This finding is similar to the results of previous studies that reported that alterations in AbdH muscle activity affected the navicular position [13,14]. This may be attributed to the important functions of the AbdH muscle maintaining the MLA height, thus, this muscle could help to prevent an increase in navicular drop [12,36]. Hahm et al. reported a significant increase in plantar flexor muscle strength resulting from a 2-week use of TENS compared with placebo TENS [37]. It is likely that TENS activated group I and II afferents, reinforcing the physiological recruitment of motor neurons and transmitting a sensory volley to the brain [38]. This finding corresponds with those of our previous study, which also revealed an improvement in NDT in response to a 4-week TENS treatment of the AbdH muscle in older adults [8]. The significance of the SFE program in the reduction of the navicular drop in the present study was similar to the results of a 5-week SFE training intervention in flexible flatfoot patients reported by Kim and Kim [39]. Mulligan and Cook [21] also reported that healthy individuals who performed SFE showed a significant change in navicular drop after 4 and 8 weeks of training. This could be explained by the muscle strength gain following AbdH muscle training observed via the NDT measurement.

Muscle size is currently considered to be one of the best determinants of muscle strength [40]. It can be easily assessed by measurement of the muscle thickness or CSA of the muscle [41]. In the present study, increasing the CSA of the AbdH muscle using TENS with SFE was similar to the findings of a previous study that showed an increase in the CSA of the quadriceps muscle after ES [42]. Moreover, ES increased the CSA of the AbdH muscle after 4 weeks of intervention [26]. The increase in muscle size using TENS may be due to stimulation of the largest-diameter muscle fibers before the smaller-diameter muscle fibers, whereas the recruitment pattern is reversed during volitional exercise, in which the smallest alpha motor neurons are recruited first [8,25]. In support of this notion, Onigbinde et al. [43] demonstrated quadricep femoris hypertrophy following 8 weeks of TENS using a frequency of 85 Hz and pulse width of 100 μs and Nishikawa et al. [44] reported increased muscle mass after lower limb muscle stimulation. However, there was no significant difference in muscle size changes between those who received TENS and SFE (mean, 10.2%) and SFE alone (mean, 20.7%) in the present study. This finding was unexpected; therefore, further studies are required using a longer period or individuals with more severe muscle weakness. A similar study design and intervention was investigated in a study by Namsawang et al. [26]. They demonstrated that high voltage pulsed current with SFE for 4 weeks was more effective for increasing the CSA of AbdH muscle by about 17.5% than SFE alone (8.1%) in adults with flat feet. However, further studies are warranted to confirm this result for the use of TENS in the elderly with flat feet.

In the present study, both groups showed a significant improvement in TUG, indicating a better balance ability. However, there was a greater improvement in TUG performance in the TENS plus SFE group compared with the SFE alone group. This may be due to the increased activation of the somatic sensory system, which is involved in the maintenance and control of the standing position, increased cerebral activity activation [39], and a direct increase in the stimulated muscular activation of both slow and fast-twitch muscles fibers in elderly patients in response to ES intervention. Based on the findings of previous studies, TENS may be used to increase muscular activation and strength as well as motor function [45]. Similar to previous findings, balance and gait performance were substantially improved in stroke patients in response to TENS [45]. Enoka et al. [46] found the increase in sensory feedback elicited by TENS produced clinically significant improvements in gait speed, walking endurance, and dynamic balance. Jang and Park also demonstrated that NMES produced a significant TUG improvement following a 4-week intervention in older adults.

The 5TSTS is widely used to assess functional mobility, functional lower limb muscle strength, balance control ability, and risk of falls in older adults [47,48]. In the present study, the TENS plus SFE group showed similar findings to a previous study that revealed an improvement in 5TSTS after the NMES of lower extremity treatment in older adults [20]. Sham SFE for 4 weeks also showed a similar improvement in the 5TSTS, which led to increased strength of the toe plantar flexor muscles, decreasing the risk of falls in older people [49]. Hence, functional mobility was also likely to be improved by 5TSTS improvement.

The fear of falling has negative effects on the physical and functional well-being of elderly people. It leads to a higher dependence status and decreased ability to perform daily living and socialization tasks. It also contributes to physical inactivity, which, in turn, leads to sarcopenia, the loss of balance, gait abnormalities, and an increased risk of falls [50]. Atrophy of the plantar intrinsic foot muscles in older adults can interfere with the ability of the postural system to maintain balance during gait [7]. Thus, stronger plantar intrinsic foot muscles should enhance balance in older adults [51]. An improved balance is associated with a decreased fear of falling. Several previous studies have reported the benefits of using ES to improve balance. We previously reported that TENS strengthened the AbdH muscle and improved the FES-I score [8]. An improvement of the intrinsic foot flexor strength efficiently enhanced standing and gait performances, reduced the risk of falling, and enhanced mobility [6,16,52,53]. Furthermore, an improved balance ability was seen after 4-week training of the AbdH muscle in older adults [8], which corresponds to the findings of the SFE group in the present study.

The present study has some limitations. First, the study did not categorize the severity of the intrinsic foot muscle weakness in each group. However, the baseline measurement of NDT did not differ between the groups, and this could be assumed to indicate homogeneity in terms of intrinsic foot muscle weakness severity between the two groups. Interestingly, we found a moderate association between NDT improvement in a relation to baseline NDT and the severity of the intrinsic foot muscle weakness only in the TENS plus SFE group (Figure 2). This suggests that TENS plus SFE treatment may have superior beneficial effects in terms of increasing muscle strength, particularly in those with higher NDT scores or flat feet in older adults. Thus, the findings of the present study should be further investigated in those with flat feet. Second, a longer training period or chronic training is recommended to achieve a clearer response to training, particularly using TENS with SFE, in terms of CSA improvement. Third, the follow-up monitoring should be necessary for further study especially when a long-term problem is concerned, such as risk of falls. Mulligan and Cook (2013) [21] claimed that the navicular drop, the arch height index, and the star excursion balance test were still improved after 8 weeks of 4-week intrinsic foot muscles training program.

## 5. Conclusions

In summary, combined treatment with TENS plus SFE is a selective intervention protocol to indirectly increase the strength of the intrinsic foot muscles, improve balance ability, and reduce the fear of falls as well as the results in using SFE alone. However, there is a superior effect on balance ability and an improvement in the risk of falls, as measured by TUG, in response to the combined intervention. Thus, older adults with impaired mobility or sarcopenia may benefit from using TENS as an alternative or adjuvant treatment for training or rehabilitation.

## Figures and Tables

**Table 1:** Physical characteristics and pre-measurements of participants of the study (*n* = 68).

	TENS + SFE (*n* = 34)	SFE Alone (*n* = 34)
Age (years)	68.6 ± 4.6	68.7 ± 4.0
Sex	6 Males, 28 females	6 Males, 28 females
Weight (Kg)	59.9 ± 11.3	60.7 ± 11.5
Height (cm)	158.6 ± 9.6	158.9 ± 8.2
BMI (Kg∙m^−2^)	23.8 ± 3.7	23.9 ± 3.1
NDT (mm)	6.01 ± 1.61	5.96 ± 3.03
FES-I (points)	27.7 ± 10.2	26.4 ± 7.1
CSA of AbdH (cm^2^)	1.93 ± 0.49	1.86 ± 0.50
5TSTS (s)	6.58 ± 1.68	7.53 ± 1.78
TUG (s)	6.98 ± 1.53	7.77 ± 1.75

BMI, body mass index; NDT, navicular drop test; FES-I, Falls Efficacy Scale International; CSA, cross sectional area; AbdH, abductor hallucis muscle; 5TSTS, 5 times sit to stand; TUG, timed up and go.

**Table 2:** Median and interquartile range of outcome measures before and after 4 weeks TENS combined SFE and SFE alone (*n* = 68).

	TENS + SFE (*n* = 34)		SFE Alone (*n* = 34)		Between Groups
	Pre	Post	Pre	Post	
NDT (mm)	5.57 (4.80, 6.81)	4.71 (4.15, 5.61) *	5.16 (3.56, 8.04)	4.60 (2.98, 4.60) *	0.64
FES-I (points)	27.0 (19.0, 36.3)	19 (16.0, 22.3) *	25.0 (20.8, 31.0)	21.0 (17.0, 25.0) *	0.83
CSA of AbdH (cm^2^)	1.86 (1.61, 2.31)	2.05(1.76, 2.53) *	1.79 (1.56, 2.20)	2.16 (1.77, 2.50) *	0.49
5TSTS (s)	6.52 (5.50, 7.87)	5.61 (4.99, 6.25) *	7.53 (6.10, 8.40)	6.10 (5.36, 7.84)*	0.68
TUG (s)	6.62 (6.15, 7.67)	5.88 (5.43, 6.49) *	7.41 (6.47, 8.90)	6.67 (6.03, 8.25) *	0.008

* indicated *p* < 0.05 the difference between pre and post 4 weeks of training. NDT, navicular drop test; FES-I, Falls Efficacy Scale International; CSA, cross sectional area; AbdH, abductor hallucis muscle; 5TSTS, 5 times sit to stand; TUG, timed up and go.

## Data Availability

The datasets used and analyzed during this study are available from the corresponding author on reasonable request.
